# Leukocyte Integrin Antagonists as a Novel Option to Treat Dry Age-Related Macular Degeneration

**DOI:** 10.3389/fphar.2020.617836

**Published:** 2021-01-29

**Authors:** Monica Baiula, Alberto Caligiana, Andrea Bedini, Junwei Zhao, Federica Santino, Martina Cirillo, Luca Gentilucci, Daria Giacomini, Santi Spampinato

**Affiliations:** ^1^ Laboratory of Cellular and Molecular Pharmacology, Department of Pharmacy and Biotechnology, University of Bologna, Bologna, Italy; ^2^ Department of Chemistry “G. Ciamician”, University of Bologna, Bologna, Italy; ^3^ Laboratory of Design and Synthesis of Biologically Active Compounds, Department of Chemistry “G. Ciamician”, University of Bologna, Bologna, Italy; ^4^ Specilization School of Hospital Pharmacy, Department of Pharmacy and Biotechnology, University of Bologna, Bologna, Italy

**Keywords:** age-related macular degeneration, retinal pigment epithelium cells, leukocyte integrins, inflammation, integrin antagonist, immune cells

## Abstract

Age-related macular degeneration (AMD) is a complex multifactorial degenerative disease that leads to irreversible blindness. AMD affects the macula, the central part of the retina responsible for sharp central vision. Retinal pigment epithelium (RPE) is the main cellular type affected in dry AMD. RPE cells form a monolayer between the choroid and the neuroretina and are in close functional relationship with photoreceptors; moreover, RPE cells are part of the blood retina barrier that is disrupted in ocular diseases such as AMD. During ocular inflammation lymphocytes and macrophages are recruited, contact RPE and produce pro-inflammatory cytokines, which play an important role in AMD pathogenesis. The interaction between RPE and immune cells is mediated by leukocyte integrins, heterodimeric transmembrane receptors, and adhesion molecules, including VCAM-1 and ICAM-1. Within this frame, this study aimed to characterize RPE-leukocytes interaction and to investigate any potentially beneficial effects induced by integrin antagonists (DS-70, MN27 and SR714), developed in previous studies. ARPE-19 cells were co-cultured for different incubation times with Jurkat cells and apoptosis and necrosis levels were analyzed by flow cytometry. Moreover, we measured the mRNA levels of the pro-inflammatory cytokine IL-1β and the expression of adhesion molecules VCAM-1 and ICAM-1. We found that RPE-lymphocyte interaction increased apoptosis and necrosis levels in RPE cells and the expression of IL-1β. This interaction was mediated by the binding of α_4_β_1_ and α_L_β_2_ integrins to VCAM-1 and ICAM-1, respectively. The blockade of RPE-lymphocyte interaction with blocking antibodies highlighted the pivotal role played by integrins. Therefore, α_4_β_1_ and α_L_β_2_ integrin antagonists were employed to disrupt RPE-lymphocyte crosstalk. Small molecule integrin antagonists proved to be effective in reducing RPE cell death and expression of IL-1β, demonstrating that integrin antagonists could protect RPE cells from detrimental effects induced by the interaction with immune cells recruited to the retina. Overall, the leukocyte integrin antagonists employed in the present study may represent a novel opportunity to develop new drugs to fight dry AMD.

## Introduction

Age-related macular degeneration (AMD) is a progressive degenerative disease that leads to irreversible blindness in elderly people (Akyol and Lotery, 2020). AMD affects the macula, the ocular region responsible for sharp central vision. Two forms of AMD have been described: dry AMD is the most common type of the disease and causes atrophic degeneration of the macula leading to gradual vision loss (advanced AMD or geographic atrophy). On the contrary, wet AMD is characterized by choroidal neovascularization (CNV) that rapidly causes blindness (Ambati and Fowler, 2012). Since vascular endothelial growth factor (VEGF) is a predominant proangiogenic factor in CNV, wet AMD can be treated with intravitreous administration of anti-angiogenic agents such as aflibercept and ranibizumab (Supuran, 2019). Conversely, there is currently no effective pharmacological treatment for “dry” AMD although novel therapeutic strategies are under development (Akyol and Lotery, 2020). The pathogenesis of dry AMD is not completely understood and involves a complex interplay among several mechanisms. Oxidative stress, together with other factors, including aging, genetic factors, phototoxicity, complement system activation, can lead to retinal pigment epithelium (RPE) degeneration and photoreceptors death, and can raise in immune response and inflammation (Strauss, 2005; Hanus et al., 2015). RPE is a monolayer of cells embedded between the choroid and the neuroretina. Normal physiological functions of RPE cells are important for outer retina homeostasis and for normal vision. One of the late events in AMD pathogenesis is RPE cell death, associated with the degradation of the overlying photoreceptors and the underlying choriocapillaris (Van Lookeren Campagne et al., 2014). In addition, RPE is a component of the blood-retina barrier (BRB) limiting the penetration of blood components to the retina and is also implicated in the recruitment of immune cells during local inflammation. During ocular inflammatory process lymphocytes and macrophages are recruited to the posterior compartment of the eye and produce pro-inflammatory cytokines, including tumor necrosis factor α (TNFα), interleukin-1β (IL-1β) and IL-6 (Cousins et al., 2004), which play an important role in AMD pathogenesis. Adhesion molecules, such as intercellular adhesion molecules-1 (ICAM-1) and vascular cell adhesion molecule-1 (VCAM-1) mediate leukocyte adhesion and recruitment to RPE. Cultured RPE cells constitutively express ICAM-1 but not VCAM-1; both adhesion molecules are upregulated after treatment with pro-inflammatory cytokines (Platts et al., 1995). Adhesion molecules are physiological ligands of integrins, heterodimeric transmembrane proteins formed by non-covalent association of α and β subunit (Tolomelli et al., 2017; Baiula et al., 2019). Notably, leukocyte integrins, including α_4_β_1_ and α_L_β_2_, mediate immune cell recruitment to inflamed tissue. Some agents targeting integrins have already been approved for clinical practice (Raab-Westphal et al., 2017) and several new ligands are presently under development.

Within this frame, in previous studies, we investigated new ligands targeting different types of integrin (De Marco et al., 2015; Tolomelli et al., 2015; Baiula et al., 2016; Baiula et al., 2020; Dattoli et al., 2018; Martelli et al., 2019). In particular, we identified MN27, SR714, and DS-70 as potent and selective leukocyte integrin antagonists able to significantly reduce integrin-mediated cell adhesion and intracellular signaling activation in a concentration-dependent manner (Baiula et al., 2016; Dattoli et al., 2018). Therefore, considering integrins as a valuable drug target, the current study was designed to elucidate the crosstalk between RPE and leukocytes *in vitro* and to characterize any potentially beneficial effects induced by integrin antagonists within this frame. To this purpose ARPE-19 cells were co-cultured with immune cells for different time points and we analyzed apoptosis and necrosis levels, adhesion molecule expression, integrin-mediated cell adhesion, intracellular signaling activation and IL-1β expression. Moreover, we investigated the effects of integrin antagonists on RPE-leukocytes interaction. We found that integrin antagonists were able to disrupt RPE-immune cell interaction leading to reduced RPE cell death. Therefore, our results open up the possibility to exploit integrin antagonists as innovative therapeutics to fight dry AMD.

## Materials and Methods

### Cell Culture and Treatments

ARPE-19 cells (American Type Culture Collection, ATCC, Rockville, MD; passages 4–7), a human spontaneously arising retinal pigment epithelia cell line, were grown in Dulbecco’s modified Eagle’s medium and Ham’s F12 medium (DMEM/F12, Life Technologies, Monza, Italy) supplemented with 10% fetal bovine serum (FBS, Life Technologies) and antibiotic-antimycotic solution (Life Technologies). Jurkat E6.1 cells (ATCC; passages 5–10) were cultured in RPMI 1640 (Life Technologies) supplemented with 2 mM glutamine, 10% FBS and antibiotic-antimycotic solution. Cells were cultured at 37°C under 5% CO_2_ humidified atmosphere.

To study ARPE-19-Jurkat cells interactions, ARPE-19 cells were seeded in 6-well plates and cultured until monolayers were formed. Then, Jurkat cells (10^6^ cells/well) were added and co-cultured with ARPE-19 cells for different incubation periods (1, 16, 24 and 48 hours). ARPE-19-Jurkat co-culture were performed in the presence of 1 mM Mn^2+^ to ensure integrin activation and high affinity ligand binding. At the end of the co-culture, immune cells were removed by washing the wells three times with PBS (phosphate buffered saline, Life Technologies) and ARPE-19 cells were detached with Trypsin/EDTA 1% solution (Lonza). Finally, cells were centrifuged separately and pelleted to be stored at -80° for further analyses.

Neutralizing antibodies anti-VCAM-1 (clone 51-10C9, cat. n.555645) or anti-ICAM-1 (clone LB-2, cat. n.559047) (both from BD Pharmingen™) were added to ARPE-19 cells at saturation concentration (10 μg/mL) for one hour before the addition of Jurkat cells; alternatively, Jurkat cells were pre-incubated with anti-α_4_ integrin (10 μg/mL, clone 44H6, cat. n. ab220, Abcam) or anti-α_L_ (clone HI111, cat. n.555381, BD Pharmingen) for 1 h before being overlaid on ARPE-19 cells. Thereafter, the co-culture was extended for 24 hours. Cells were collected separately and stored as above described.

Integrin antagonists employed in this study have been previously investigated (Baiula et al., 2016; Dattoli et al., 2018). These compounds had displayed a strong antagonist activity against α_L_β_2_ (MN27) or α_4_β_1_ (DS-70 and SR714) integrins (Table 1). A stock solution (10^−2^ M) of integrin antagonists was prepared in dimethyl sulfoxide (DMSO, vehicle) and its final concentration did not exceed 0.1%; an equal volume of vehicle was added as control to vehicle cells. Cells were pre-incubated with different concentrations (1–100 nM) of integrin antagonists for 30 min at 37°C before adding Jurkat to ARPE-19 cells for 24 hours.

**TABLE 1:** Solid-phase, SPA binding and inhibition of cell adhesion of integrin antagonists MN27, DS-70 and SR714 to VCAM-1 or ICAM-1 (2 μg/mL) (Baiula et al., 2016; Dattoli et al., 2018).

Integrin antagonists	Solid-phase binding/SPA^a^ IC_50_ (nM)	Cell adhesion^b^ Jurkat, α_4_β_1_/VCAM-1 IC_50_ (nM)	Cell adhesion^b^ Jurkat, α_L_β_2_/ICAM-1 IC_50_ (nM)
MN27 (α_L_β_2_)	6.7 ± 2.5 (α_L_β_2_)	574.0 ± 1.7	0.39 ± 0.02
DS-70 (α_4_β_1_)	8.3 ± 3.2 (α_4_β_1_)	5.04 ± 0.51	>5000
SR714 (α_4_β_1_)	1.1 ± 0.1 (α_4_β_1_)	1.39 ± 0.04	>5000

^a^ IC_50_ values of β-lactam compounds and DS-70 on leukocyte integrins determined by a competitive solid-phase binding assay to specific ligand (ICAM-1 for α_L_β_2_) or by scintillation proximity assay (SPA, FN for α_4_β_1_). Six independent experiments were run in quadruplicate. Data are expressed as means ± SD (Baiula et al., 2016; Dattoli et al., 2018).

^b^ In a cell-based assay, the adhesion of a cell line preferentially expressing a specific integrin heterodimer to an immobilized adhesion molecule was measured. Six independent experiments were run in quadruplicate. Data are expressed as means ± SD (Baiula et al., 2016; Dattoli et al., 2018).

### Adhesion Assay

ARPE-19 cells were plated in black 96-well plates (15000 well) and grown for 24–48 h. ARPE-19 cells were pretreated with or without anti-ICAM-1 or anti-VCAM-1 antibodies (10 μg/mL) for 1 h. Jurkat cells (50000 cells/well) were labelled with Cell Tracker green CMFDA (12.5 μM, 30 min at 37°C, Life Technologies) and, after three washes with PBS, were overlaid on ARPE-19 cells; the cells were incubated at 37°C for 3 h. Then, after three gently washes with PBS to remove nonadherent Jurkat cells, the remaining cells were lysed with 0.5% Triton X-100 in PBS (for 30 min at 4°C). The number of fluorescently labeled adherent Jurkat cells was determined measuring fluorescence (Ex485 nm/Em535 nm) in an EnSpire Multimode Plate Reader (PerkinElmer, Waltham, MA, USA). The number of adherent cells was determined by comparison with a standard curve built up in the same plate with known numbers of labelled Jurkat cells. To investigate the role of integrins in ARPE-19-Jurkat interactions, Jurkat cells were pre-incubated with 10 μg/mL of anti-α_4_β_1_ integrin or anti-α_L_ antibody for 1 h. In another set of experiment, Jurkat cells were exposed to increasing concentrations (1–100 nM) of integrin antagonists (DS-70, MN27 or SR714) for specified times.

### Apoptosis Detection

Apoptosis was analyzed by flow cytometry as previously described (Baiula et al., 2016; Dattoli et al., 2018). Phycoerythrin-conjugated annexin V (annexin V-PE) and 7-amino-actynomicin D (7-AAD; Guava Nexin Reagent, Millipore, Darmstadt, Germany) were employed to determine the percentage of viable, early apoptotic and late apoptotic/necrotic cells by flow cytometry. ARPE-19 and Jurkat cells were co-cultured for different time points and then cells were collected separately by centrifugation and resuspended in 100 μL of complete medium. For apoptosis/necrosis staining, 100 μL of Nexin reagent were added and ARPE-19 and Jurkat cells were incubated for 20 min at room temperature in the dark, following manufacturer’s instructions. Thereafter, cells were analyzed on a Guava EasyCyte 5 flow cytometer (Millipore); at least 10000 cells/sample were acquired. Three populations of cells can be identified by this assay: viable cells (annexin V-PE and 7-AAD negative), early apoptotic cells (annexin V-PE positive and 7-AAD negative), and late stage apoptosis or necrotic cells (annexin V-PE and 7-AAD positive).

### Flow Cytometry

To evaluate expression of integrins and adhesion molecules on ARPE-19 cells, anti-ICAM-1, anti-VCAM-1, anti-α_5_ (clone VC5, cat. n.555651, BD Pharmingen), anti-α_M_ (clone M1/70, cat. n. MAB1387Z, Chemicon International), anti-α_L_ (clone HI111, cat. n.555381, BD Pharmingen), anti-β_1_ (clone HUTS-21, cat. n. 556049, BD Pharmingen), anti-β_2_ (clone 6.7, cat. n. 555923, BD Pharmingen), or anti-α_4_ monoclonal antibody (cat. n. ab202969, Abcam), were used in 1% BSA/HBSS (Hanks’ Balanced Salt Solution, Life Technologies) for 45 min at 4°C. After two washes in 1% BSA/HBSS cells were incubated with goat anti-rabbit IgG (H + L) secondary antibody, Alexa Fluor 488 in 1% BSA/HBSS (Life Technologies) for 45 min at 4°C. After two washes with 1% BSA/HBSS, cells were resuspended in PBS and analyzed at Guava® EasyCyte™ flow cytometer (Millipore) and at least 10000 cells/sample were analyzed. Evaluation of the relative fluorescence for nonspecific binding by exposing the cells to an isotype control monoclonal antibody FITC mouse IgG (BD Pharmingen) was done for data normalization (Dattoli et al., 2018).

### Real Time RT-PCR

At the end of the co-culture, ARPE-19 and Jurkat cells were collected separately as described above and centrifuged (500 g for 5 min). PD 98059 (10 μM, 30 min; Sigma Aldrich), a non-competitive blocker of ERK1/2 activation (Di Toro et al., 2005; Baiula et al., 2012) was used to investigate the role of integrin-mediated ERK1/2 signaling. Total cellular RNA was extracted with Trireagent® (Sigma-Aldrich, Milan, Italy) and digested with Rnase-free Dnase (Thermo Fisher Scientific, Walthman, MA, USA) for 15 min at 25°C. High Capacity cDNA reverse transcription kit (Life Technologies) was used to reverse transcribe a 2-µg sample, according to the manufacturer’s instructions. Relative quantification of human IL-1β transcripts was performed by Real-Time PCR using the StepOne Instrument (Life Technologies). The GoTaq® qPCR master mix (Promega, Madison, Wisconsin, USA) was chosen to perform the reaction as follow: denaturation at 95°C for 10 min, followed by 40 cycles of 95°C for 30 sec, 68°C for 30 sec and 72°C for 30 sec. To amplify human IL-1β cDNA, a sense primer (5′-CAA​GGG​CTT​CAG​GCA​GGC​CG-3′) and an antisense primer (5′-TGA​GTC​CCG​GAG​CGT​GCA​GT-3′) were used at 0.25 µM concentration for producing a 213-bp fragment (261–474 bp; GenBank Accession no. NM_000576.2). As a control, a 169-bp fragment of the human L19 ribosomal protein gene (62–230 bp; GenBank Accession No. BC062709) was amplified with a sense primer (5′-CTA​GTG​TCC​TCC​GCT​GTG​G-3′) and an antisense primer (5′-AAG​GTG​TTT​TTC​CGG​CAT​C-3′) at 0.25 µM concentration (Bedini et al., 2017). No-template controls and DNA melting curve analysis were employed as controls. Relative expression of RT-PCR products was determined using the ΔΔC_t_ method (Winer et al., 1999). Each sample was run in triplicate and L19 was chosen for normalization because of its consistent expression relative to other housekeeping genes among the experimental groups in our study.

### Western Blot Analysis

Western blot analysis was performed as previously described (Dattoli et al., 2018) with the following modifications. Briefly, ARPE-19 cells were plated into 60 mm dishes until a confluence of 80–90% was reached; the cells were incubated with Jurkat cells (10^6^ cells/well) as described in a previous section. Thereafter, the cells were homogenized in lysis buffer (50 mM Tris-HCl, 300 mM NaCl, 1 mM EDTA, 1 mM Na_3_VO_4_, 1 mM NaF, 10% glycerol, 1%Triton-X 100) supplemented with phosphatase inhibitor and protease inhibitor cocktail (both 1:100, Sigma-Aldrich SRL). The homogenates were sonicated for 10 sec and then centrifuged at 17000 g for 25 minutes at 4°C; protein concentration was determined by BCA assay (Pierce, Rockford, IL, USA). Protein extracts (15 µg) were denatured at 95°C for 3 min and separated by 12% SDS-PAGE. The membranes were blocked in 5% BSA in TBS-T (20 mM Tris-HCl, pH 7.5, 137 mM NaCl, 0.1 (v/v) % Tween 20) for 1 h and incubated with anti-phospho-ERK 1/2 (extracellular signal-regulated kinase 1/2, 1:1000; Cell Signaling Technology, Danvers, MA, USA) or anti-total ERK1/2 antibodies (1:1000; Cell Signaling Technology) overnight at 4°C. Subsequently, the membranes were incubated with peroxidase-conjugated anti-rabbit secondary antibodies at a 1:8000 dilution (Santa Cruz Biotechnology, Dallas, Texas, USA) at room temperature for 1.5 h. Digital images were acquired and analyzed according to a previously reported method (Bedini et al., 2008). Experiments were replicated independently at least four times.

### Statistical Analysis

All data are presented as the mean ± SD for the number of experiments reported. Statistical comparisons were performed using one-way ANOVA followed by Newman-Keuls test (GraphPad Prism, version 5.0; GraphPad Software, Inc., La Jolla, CA, USA). Statistical significance is stated as *p* < 0.05.

## Results

### Characterization of ARPE-19-Jurkat cells Interaction

**Figure 1:**
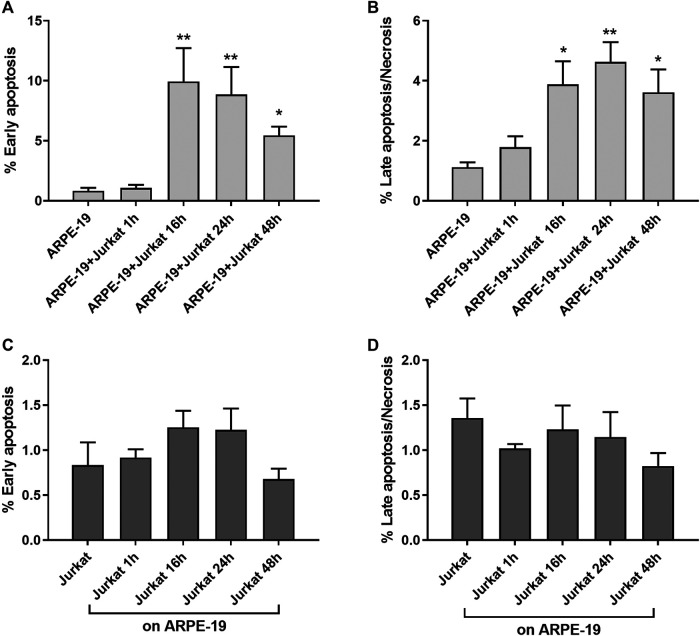
ARPE-19-Jurkat cells co-culture resulted in increased apoptosis and necrosis. Interactions between ARPE-19 and Jurkat cells induced a significant increase of apoptosis **(A)** and necrosis **(B)** in ARPE-19 cells after 16 h of co-culture and up to 48 h. No significant changes in apoptosis **(C)** or necrosis **(D)** was observed in Jurkat cells incubated with ARPE-19 for different time points (0–48 h). Cells not co-cultured were considered as reference (shown as ARPE-19 or Jurkat in the figure). Apoptosis and necrosis were measured by flow cytometry and the results are presented as the percentage of early apoptotic and late apoptotic/necrotic cells. Values are mean ± SD from four experiments conducted in triplicate. *p < 0.05; **p < 0.01 vs ARPE-19/Jurkat (Newman-Keuls test after ANOVA).

Infiltration of mononuclear cells in the outer blood–retina barrier has been associated to several retinal diseases, including AMD (Gehrs et al., 2006). Moreover, RPE-to-immune cells contact and RPE apoptosis/necrosis were observed within AMD lesions (Ge-Zhi et al., 1996). Key mechanisms of RPE loss during AMD are both apoptosis and necrosis (Yang et al., 2011; Hanus et al., 2015). To evaluate the effects of RPE-immune cells interaction on apoptosis and necrosis, Jurkat cells were overlaid on ARPE-19 cells for different time points (1, 16, 24 and 48 h); thereafter, immune cells were removed by washing with PBS and collected by centrifugation, whereas ARPE-19 cells were detached by trypsinization and then centrifugated. Apoptosis/necrosis levels were evaluated by Annexin V assay as described in the methods section. Interactions between ARPE-19 and Jurkat cells led to a significant increase of apoptosis and necrosis in ARPE-19 cells (Figure 1). Increment in apoptosis and necrosis in ARPE-19 cells were observed after 16 h of co-culture and maintained up to 48 h. As regards immune cells, co-cultured with RPE for different incubation times, no significant changes of apoptosis or necrosis were found, as shown in Figure 1 (panels C and D). Representative apoptosis/necrosis cytograms are shown in Supplementary Figure S1.

**Figure 2:**
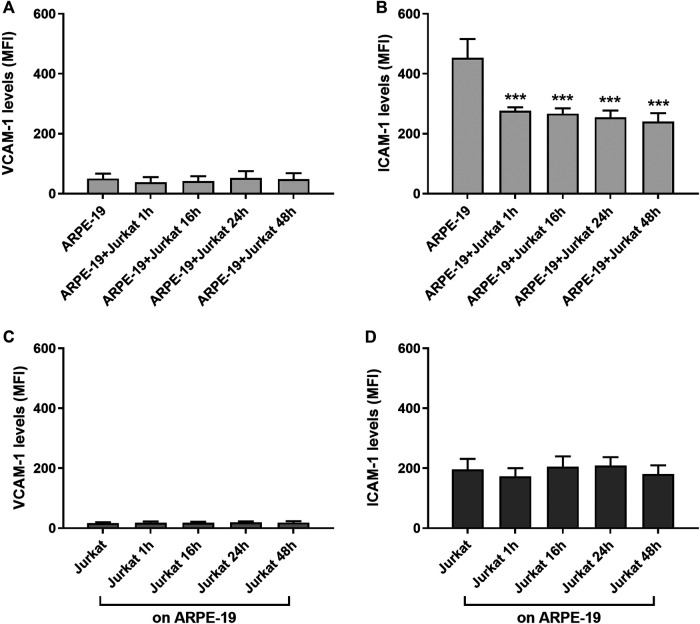
Evaluation of ICAM-1 and VCAM-1 expression levels on ARPE-19 and Jurkat cells co-cultured for different time points (0–48 h). VCAM-1 expression was not influenced in both ARPE-19 **(A)** and Jurkat **(C)** cells. The interactions between ARPE-19 and Jurkat cells induced a significant reduction of ICAM-1 levels expressed on ARPE-19 cells at all time point considered **(B)**, whereas no changes were measured in ICAM-1 levels on Jurkat cells **(D)**. Data are expressed as mean fluorescence intensity (MFI) ± SD of four independent experiments carried out in triplicate. MFI values for respective isotype control mAb were set to 0. ***p < 0.001 versus ARPE-19 (Newman−Keuls test after ANOVA).

Adhesion molecules, such as ICAM-1 and VCAM-1, are endothelial- and leukocyte-associated membrane receptors playing a pivotal role in cell-cell interactions and in leukocyte transmigration across the endothelium. Both ICAM-1 and VCAM-1 are expressed on RPE cells (Holtkamp et al., 2001; Zhu et al., 2016) and can mediate RPE-leukocyte interaction. Thus, to determine the role of adhesion molecules in RPE-immune cells interaction in our *in vitro* model, expression levels of ICAM-1 and VCAM-1 were evaluated in both ARPE-19 and Jurkat cells at different co-culture intervals (1, 16, 24 and 48 h) by flow cytometry. In ARPE-19 cells VCAM-1 displayed low basal expression levels that were not altered at any of the considered time points following co-culture with Jurkat cells (Figure 2A). On Jurkat cells, expression of VCAM-1 expression was very low and was not significantly modified by interaction with ARPE-19 cells (Figure 2C). Similarly, we did not observe any changes in ICAM-1 levels on Jurkat cells after co-culturing them with ARPE-19 cells, although this adhesion molecule displayed higher basal expression levels on Jurkat cells as compared to VCAM-1 (Figure 2D). On the contrary, ARPE-19 cells displayed very high levels of ICAM-1 on their cell membrane; notably, ICAM-1 expression in ARPE-19 was significantly decreased by the interaction with Jurkat cells starting the effect after 1 h of co-culture and lasting up to 48 h of ARPE-19-Jurkat co-culture (Figure 2B). Representative histograms of VCAM-1 and ICAM-1 levels in both ARPE-19 and Jurkat cells are shown in Supplementary Figure S2.

**Figure 3:**
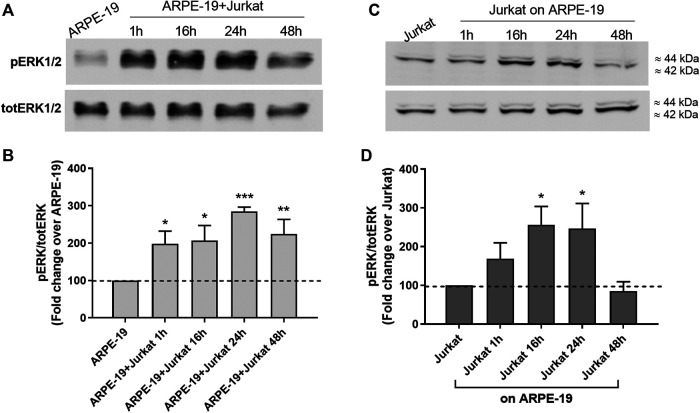
Co-culture of RPE and immune cells induced activation of intracellular signaling leading to the phosphorylation of ERK1/2. ERK1/2 activation was observed soon after 1 h of co-culture in ARPE-19 cells and was maintained up to 48 h **(A-B)**; on the contrary, in Jurkat cells ERK1/2 phosphorylation was significantly increased after 16 h and 24 h of co-culture **(C-D)**. Representative Western blot images are shown **(A and C)** and the semiquantitative densitometric analysis of the bands **(B and D)** is represented in the graphs (mean ± SD of four independent experiments); the amount of pERK1/2 is normalized to that of totERK1/2. *p < 0.05; **p < 0.01; ***p < 0.001 versus ARPE-19/Jurkat (Newman−Keuls test after ANOVA).

MAPK ERK1/2 signaling is one of the most widely investigated intracellular pathway with regard to integrins and adhesion molecules (Hubbard and Rothlein, 2000; Cox et al., 2006; Meldolesi, 2016). Therefore, we analyzed ERK1/2 activation during ARPE-19-Jurkat co-culture. As shown in Figure 3, we observed a significant increase of ERK1/2 phosphorylation, evidence of ERK1/2 signaling activation, both in ARPE-19 and in Jurkat cells throughout co-culture duration. The interaction between RPE and immune cells led to a fast activation of ERK1/2 in ARPE-19 cells: accordingly, ERK1/2 phosphorylation increment was observed after 1 h of co-culture and was sustained up to 48 h (Figure 3A,B). In Jurkat cells, phosphorylation of ERK1/2 was significantly increased after 16 and 24 h of co-culture with ARPE-19 cells (Figures 3C,D).

**Figure 4:**
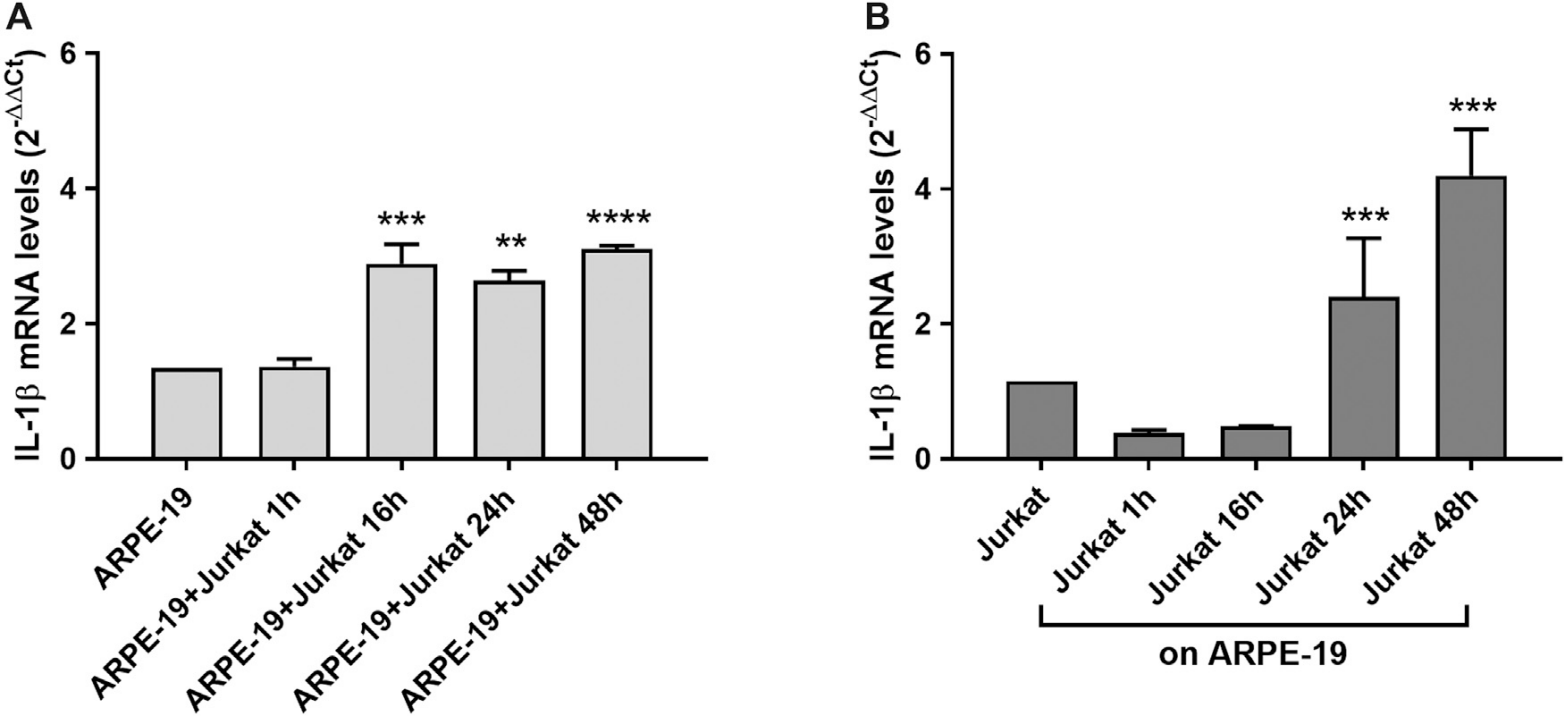
Effects of ARPE-19-Jurkat cells co-culture on IL-1β evaluated as mRNA levels. IL-1β expression was significantly increased in both ARPE-19 **(A)** and Jurkat **(B)** cells after 16 h and 24 h of co-culture, respectively. The increment of IL-1β levels was maintained up to 48 h of co-culture. Data are expressed as mean ± SD of four independent experiments carried out in triplicate. **p < 0.01; ***p < 0.001; ****p < 0.0001 versus ARPE-19/Jurkat (Newman−Keuls test after ANOVA).

Increased levels of inflammatory cytokines are detected in the eye of patients affected by AMD (Jonas et al., 2012). IL-1β is a pro-inflammatory cytokine that can be produced and released from different cells types. To evaluate changes in IL-1β expression during RPE-immune cells interaction, Jurkat cells were overlaid on ARPE-19 cells and co-cultured for different incubation times (1, 16, 24, 48 h). We observed a significant increment of IL-1β mRNA levels in ARPE-19 cells after 16 h and up to 48 h of co-culture with Jurkat cells (Figure 4A). In addition, ARPE-19-Jurkat interactions induced increased levels of IL-1β also in Jurkat cells after 24 and 48 h of co-culture (Figure 4B).

### Integrins and Adhesion Molecules are Involved in ARPE-19-Jurkat Cell Interactions

**Figure 5:**
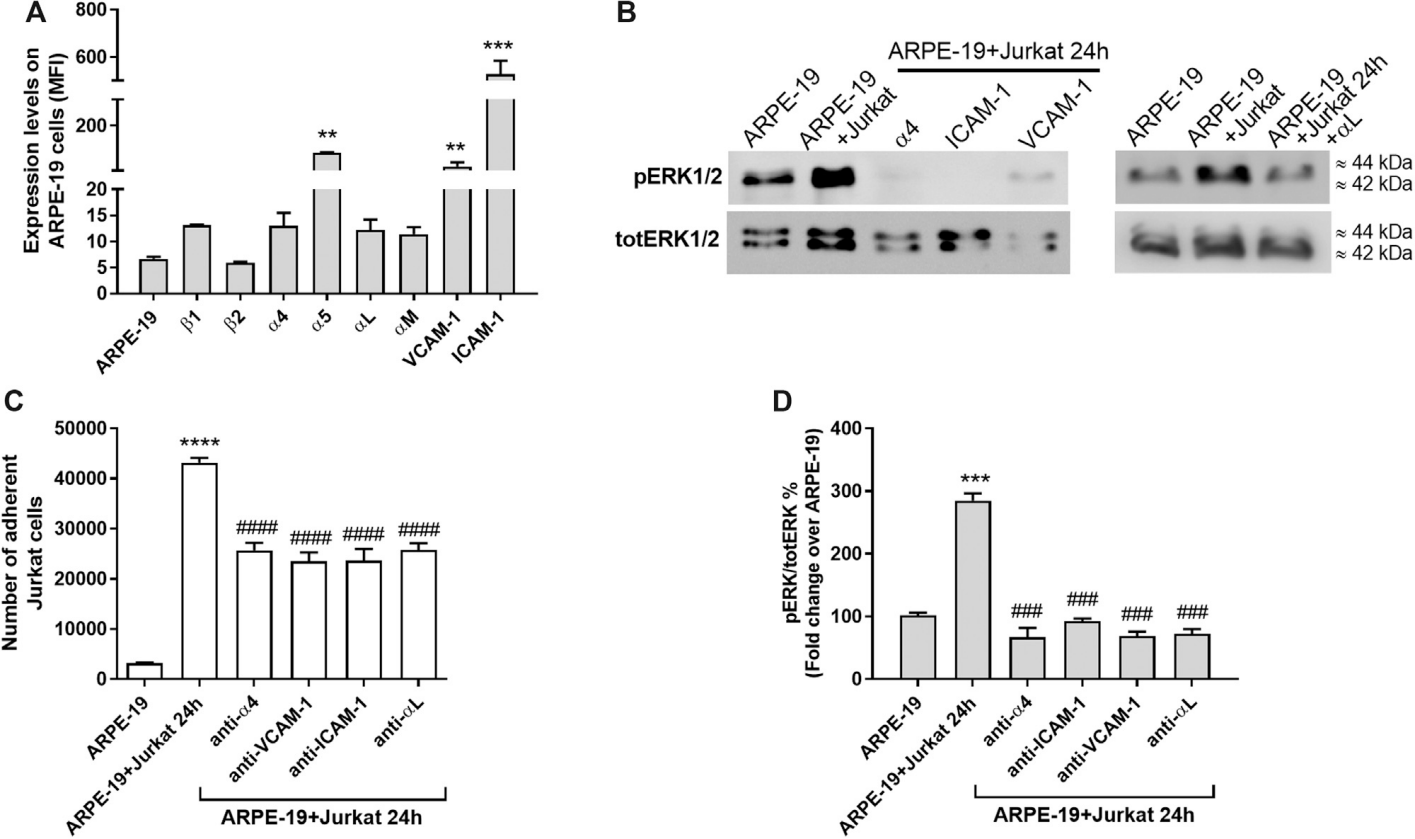
Integrins and adhesion molecules involvement during co-culture of ARPE-19-Jurkat cells **(A)** Analysis of integrin expression on ARPE-19 cells evaluated by flow cytometry. α_5_ integrin, VCAM-1 and ICAM-1 are significantly expressed on ARPE-19 cells; on the contrary ARPE-19 cells express low levels of α_4_, α_L_, α_M_, β_1_ and β_2_ integrins. Data are expressed as mean fluorescence intensity (MFI) ± SD of four independent experiments carried out in triplicate. MFI values for respective isotype control monoclonal antibody (mAb) were set to 0. Representative histograms are shown in Supplementary Figure 3. (B) Blocking VCAM-1 or ICAM-1, expressed on ARPE-19 cells, or α_4_ integrin, expressed on Jurkat cells, using specific mAbs, significantly reduced the number of Jurkat cells adhered to ARPE-19 cells. Data are expressed as mean ± SD of four independent experiments carried out in quadruplicate **(C–D)** The blockade of VCAM-1 or ICAM-1, expressed on ARPE-19 cells, or α_4_ integrin, expressed on Jurkat cells, using specific mAbs, prevented the activation of integrin-mediated intracellular signaling in ARPE-19 cells after 24 h of co-culture with Jurkat cells. A representative western blot image is shown **(C)** and the semiquantitative densitometric analysis of the bands **(D)** is represented in the graph (mean ± SD of four independent experiments); the amount of pERK1/2 is normalized to that of totERK1/2. **p < 0.01; ***p < 0.001; ****p < 0.0001 vs ARPE-19; ###*p* < 0.001 vs ARPE-19 + Jurkat 24 h (Newman−Keuls test after ANOVA).

In previous studies the expression of integrins and adhesion molecules on RPE has been described (Holtkamp et al., 2001; Elner et al., 2003; Bian et al., 2018); therefore, to confirm those results in our *in vitro* model, integrin expression on ARPE-19 cells was quantified by flow cytometry. High levels of α_5_ integrin, VCAM-1 and ICAM-1 were observed for ARPE-19 cells (Figures 2, 5A; see also Supplementary Figure S3). On the contrary, only low amounts of α_4_, α_L_, α_M_, β_1_, β_2_ subunits were detectable on the surface of ARPE-19 cells (Figure 5A). Jurkat cells are considered a suitable cell model to study integrins and integrin-mediated cell adhesion and they express mainly α_4_β_1_ and α_L_β_2_ integrins (Tolomelli et al., 2015; Dattoli et al., 2018). To demonstrate the important role played by integrins and adhesion molecules into the cross-talk between RPE and leukocytes, Jurkat cells were pre-treated with a neutralizing anti-α_4_β_1_ or anti-α_L_β_2_ integrin antibodies, whereas ARPE-19 cells were pre-incubated with anti-ICAM-1 or anti-VCAM-1 blocking antibody for 1 h. All the antibodies were added at saturating concentration (10 μg/mL). After pre-treating cells with the above listed antibodies, ARPE-19 and immune cells were co-cultured for 24 h; afterwards, cells were collected for further analysis. Integrins and adhesion molecules expressed on ARPE-19 and Jurkat cells strongly mediated cell adhesion as shown Figure 5B. Neutralizing antibodies against adhesion molecules (VCAM-1 and ICAM-1) or α_4_ and α_L_ integrin significantly reduced Jurkat cell adhesion to ARPE-19 cells (Figure 5B). Moreover, blocking adhesion molecules or integrins using mAbs led to the disruption of ARPE-19-Jurkat interactions and to the blockade of integrin-mediated signaling activation (Figure 5). Accordingly, neutralizing antibodies against VCAM-1, ICAM-1 or α_4_ and α_L_ were able to prevent ERK1/2 phosphorylation induced by ARPE-19-Jurkat interaction within 24 h-long co-culture. Control experiments showed that mAbs were not able to activate ERK1/2 signaling pathway in ARPE-19 cells not cultured together with Jurkat cells (Supplementary Figure S4). Overall, these data demonstrate the important role played by integrins and adhesion molecules in the interactions between RPE and immune cells.

### Integrin Antagonists Could Disrupt ARPE-19-Jurkat Interactions Via the Modulation of Adhesion, Apoptosis/Necrosis, MAPK Signaling Pathway and IL-1β Expression

In previous studies we developed several ligands targeting different types of integrin (De Marco et al., 2015; Tolomelli et al., 2015; Baiula et al., 2016, Baiula et al., 2020; Dattoli et al., 2018; Martelli et al., 2019). From the screening of a small library of β-lactam derivatives that were specifically designed by a structure-based strategy to target RGD-binding or leukocyte integrin we obtained selective and potent integrin ligands (Baiula et al., 2016). MN27 (compound 15 in (Baiula et al., 2016)), which contains an aniline basic moiety, is a very potent and selective α_L_β_2_ integrin antagonist, able to reduce significantly α_L_β_2_-mediated cell adhesion and integrin-mediated intracellular signaling pathway. SR714 (compound 4 in (Baiula et al., 2016)), an azetidinone with a short carboxylic acid terminus, selectively binds to α_4_β_1_ integrins and acts as an antagonist in cell adhesion assays and intracellular signaling evaluation (Table 1). In another study, we identified the compound named DS-70 that possesses a linear sequence completed by o-methylphenylureaphenylacetyl (MPUPA) and a simple glycine (Dattoli et al., 2018). The β-amino acid in its structure contributed to stable conformation of the overall structure and to increased serum stability. Through pharmacological characterization, DS-70 was recognized as an α_4_β_1_ antagonist with nanomolar potency: it prevented integrin-mediated cell adhesion and antagonized VCAM-1-induced degranulation of mast cells and eosinophils *in vitro*. Moreover, in a dose-dependent manner, DS-70 significantly reduced the clinical symptoms of allergic conjunctivitis as well as conjunctival levels of chemokines and cytokines in a guinea pig model of allergic conjunctivitis (Dattoli et al., 2018).

**Figure 6:**
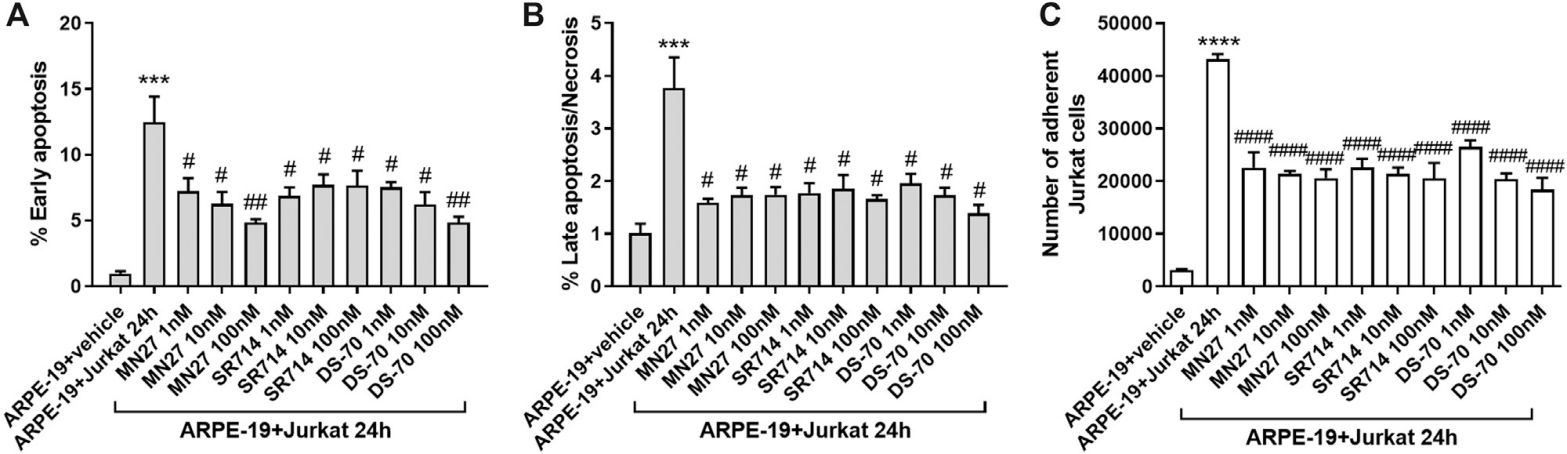
Integrin antagonists MN27, SR714 and DS-70 (1–100 nM) prevented apoptosis **(A)** and necrosis **(B)** in ARPE-19 cells induced by 24 h of co-culture with Jurkat cells. Cells not co-cultured and treated with the vehicle (DMSO) used to dissolve integrin antagonists were considered as reference (shown as ARPE-19 + vehicle in the figure). Apoptosis and necrosis were measured by flow cytometry and the results are presented as the percentage of early apoptotic cells and late apoptotic/necrotic cells. Values are mean ± SD from four experiments conducted in triplicate using different cell cultures **(C)** Integrin antagonists were also able to significantly reduce Jurkat adhesion to ARPE-19 cells. Data are expressed as mean ± SD of four independent experiments. ***p < 0.001 vs ARPE-19; #*p* < 0.05; ##*p* < 0.01; ###*p* < 0.001 vs ARPE-19 + Jurkat 24 h (Newman-Keuls test after ANOVA).

**Figure 7:**
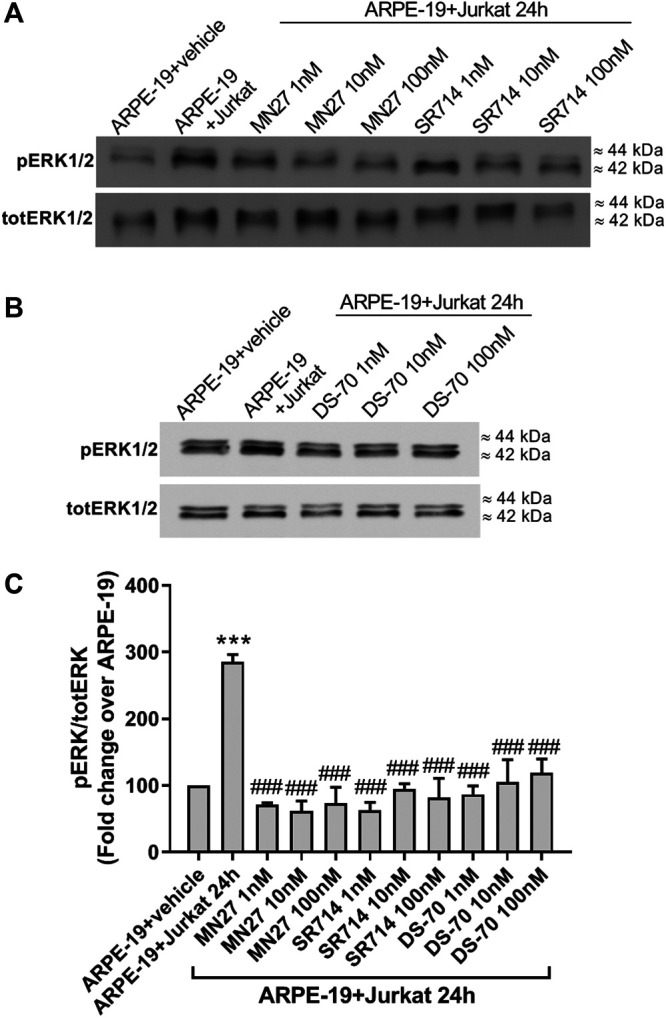
Integrin antagonists MN27, SR712 and DS-70 (1–100 nM) were able to significantly prevent integrin-mediated activation of intracellular signaling in ARPE-19 after 24 h of co-culture with Jurkat cells. ERK1/2 phosphorylation was induced during ARPE-19-Jurkat cells co-culture; on the contrary, integrin antagonists significantly reduced ERK1/2 activation. Representative western blot images are shown **(A-B)** and the semiquantitative densitometric analysis of the bands **(C)** is represented in the graphs (mean ± SD of four independent experiments); the amount of pERK1/2 is normalized to that of totERK1/2. ***p < 0.001 vs ARPE-19; ###*p*< 0.001 vs ARPE-19 + Jurkat 24 h (Newman−Keuls test after ANOVA).

To evaluate the possibility of exploiting integrin antagonists as novel agents to treat dry AMD, MN27, SR714 and DS-70 were employed in ARPE-19-Jurkat cells co-culture. Jurkat cells were pre-treated with different concentrations of MN27, SR714 or DS-70 (1–100 nM) for 30 min to block α_L_β_2_ or α_4_β_1_ integrin respectively. Thereafter, Jurkat cells were overlaid on ARPE-19 and co-cultured for 24 h; subsequently, ARPE-19 and Jurkat cells were collected separately and analyzed. As shown in Figure 6, integrin antagonists were able to significantly reduce apoptosis levels in ARPE-19 cells co-cultured with Jurkat cells for 24 h. Moreover, we found that the effect of DS-70 and MN27 was concentration-dependent (Figure 6A and Supplementary Figure S5). Integrin antagonists induced also a significant reduction of necrosis levels after disrupting the interactions between ARPE-19 and Jurkat cells. In contrast, we did not observe any change in apoptosis or necrosis levels in Jurkat co-cultured with ARPE-19 cells after the exposure to integrin antagonists (data not shown). Regarding ARPE-19-Jurkat cell adhesion, it was significantly reduced by pre-administering integrin antagonists: blocking integrins expressed on ARPE-19 or Jurkat cells, MN27, SR714 or DS-70 strongly diminished Jurkat cell adhesion to ARPE-19 cells (Figure 6C). As control experiments, ARPE-19 cells were exposed to the vehicle employed to dissolve integrin antagonists for 24 h; the vehicle, a volume corresponding to the highest antagonist concentration, did not induce apoptosis and necrosis in ARPE-19 cells, did not influence Jurkat cell adhesion to ARPE-19 cells and did not activate ERK1/2 intracellular signaling pathway in ARPE-19 cells (Supplementary Figure S6). The effects of integrin antagonists were also investigated with regard to integrin-mediated intracellular signaling activation. We found that MN27, SR714 and DS-70 fully reverted the increase of ERK1/2 phosphorylation induced during ARPE-19-Jurkat cell co-culture (Figure 7). It is intriguing to note that concentration-dependent effect induced by integrin antagonists on intracellular signaling, seen in Jurkat cells in previous studies (Baiula et al., 2016; Dattoli et al., 2018), was not observed in ARPE-19 cells after the interaction with Jurkat cells exposed to different concentrations of DS-70, MN27 or SR714.

**Figure 8:**
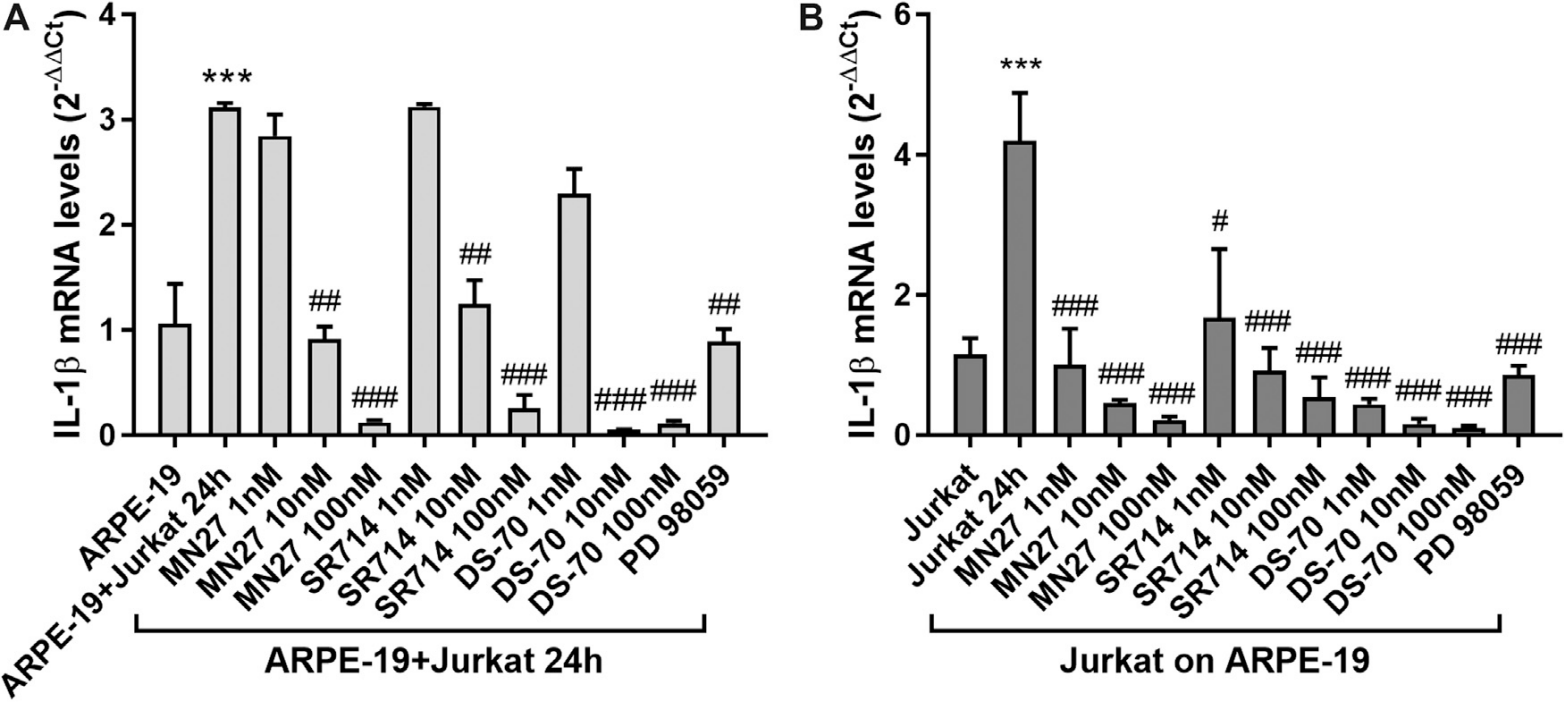
Effects of integrin antagonists on IL-1β expression in ARPE-19-Jurkat cells after 24 h of co-culture with Jurkat cells. IL-1β expression, significantly increased in both ARPE-19 **(A)** and Jurkat cells **(B)** after 24 h of co-culture, was significantly reduced by integrin antagonists MN27, SR714 and DS-70 in both ARPE-19 and Jurkat cells. PD 98059 (10 μM), an ERK1/2 signaling inhibitor pre-incubated with the cells 30 min before starting the co-culture, prevented IL-1β expression increment both in ARPE-19 and Jurkat cells. Data are expressed as mean ± SD of four independent experiments. ***p < 0.001 versus ARPE-19/Jurkat; #*p* < 0.05; ##*p* < 0.01; ###*p* < 0.001 vs ARPE-19 + Jurkat 24 h/Jurkat 24 h (Newman−Keuls test after ANOVA).

In addition, we observed that integrin antagonists prevented the co-culture-induced increase in IL-1β expression both in ARPE-19 and Jurkat cells induced by co-culture incubation (Figure 8); this effect is concentration-dependent, especially in ARPE-19 cells, in which the lowest concentration (1 nM) did not exert any effect on IL-1β levels. The noncompetitive blocker of ERK kinase PD 98059 (10 μM), added 30 min before overlaying Jurkat to ARPE-19 cells, significantly reduced the co-culture-induced up-regulation of IL-1β mRNA in both cell types (Figure 8). Thus, confirming that ERK1/2 signaling downstream of integrin activation is required for the up-regulation of IL-1β mRNA.

## Discussion

In the retina there are two types of BRB: the inner barrier comprises tight junctions of endothelial cells, whereas the outer barrier is formed by tight junctions of RPE cells. Therefore, RPE cells, together with retinal vasculature, limit the entrance of blood component to the retina. Disease conditions, including AMD (Miller et al., 1986), can lead to disruption of BRB allowing inflammatory cells, platelets and macromolecules to encounter RPE cells. In previous studies contacts between RPE and mononuclear phagocyte (Killingsworth et al., 1990; Van Der Schaft et al., 1993; Penfold et al., 2001) or T cells have been described (Liversidge et al., 1990; Devine et al., 1996). Moreover, RPE cells secrete a multitude of cytokines and chemokines (Ablonczy et al., 2011; Juel et al., 2012), contributing to the modulation of retinal inflammation. In the present study we have characterized the crosstalk between lymphocytes and RPE cells. We observed that the interaction between ARPE-19 and Jurkat cells may induce ARPE-19 cells apoptosis and necrosis, suggesting a potential role of inflammatory cells recruited at the BRB in RPE disruption observed in retinal disease conditions. It was previously demonstrated that RPE death can be due to necroptosis (Hanus et al., 2015) and that preventing this phenomenon could be beneficial for patients suffering from AMD. In addition, we found an increased expression of IL-1β in both RPE cells and lymphocytes after 16 and 24 h of co-culture, respectively. These data suggest that the physical interaction between RPE and lymphocytes could trigger and sustain an inflammatory reaction mediated by elevated levels of pro-inflammatory mediators produced by both cell types. IL-1β has important homeostatic functions but is also implicated in pathophysiological changes that occur during inflammatory disease conditions. As a matter of fact, IL-1β can be considered as a marker of inflammation (Lopez-Castejon and Brough, 2011) and is known to be involved in AMD pathogenesis. Accordingly, proinflammatory cytokines, including IL-1 β, maybe partly responsible of RPE death and photoreceptor degeneration (Kauppinen et al., 2012; Wang and Hartnett, 2016). In the eye of patients with AMD, augmented levels of inflammatory cytokines and chemokines have been reported in peripheral blood circulation (Jonas et al., 2010, Jonas et al., 2012). Moreover, previous studies showed that both immune cells (Doyle et al., 2012) and RPE cells (Fukuoka et al., 2003; Kauppinen et al., 2012) are able to produce IL-1β.

Furthermore, to better characterize RPE cell-lymphocyte interaction, we evaluated VCAM-1 and ICAM-1 levels, being these two adhesion molecules involved in immune cells recruitment to inflamed tissues. After ARPE-19-Jurkat co-culture, ICAM-1 expression was significantly reduced in ARPE-19 cells, whereas VCAM-1 levels were unaffected. VCAM-1 and ICAM-1 are endogenous ligands for α_4_β_1_ and α_L_β_2_ integrin, respectively. Previous studies have shown that the binding between adhesion molecules and integrins may contribute to the interaction between RPE and immune cells (Mesri et al., 1994; Devine et al., 1996; Holtkamp et al., 2001). The data reported in the present study confirm the contribution of integrins in immune cells recruitment to RPE. Accordingly, the adhesion of Jurkat cells to ARPE-19 is mediated by both α_4_β_1_ and α_L_β_2_ integrins and subsequently we observed integrin-mediated intracellular signaling activation. These results were confirmed by using blocking antibodies directed against α_4_β_1_, α_L_β_2_ integrins, or adhesion molecules VCAM-1 or ICAM-1. Blocking antibodies were able to reduce Jurkat cell adhesion to ARPE-19 and prevent the activation of integrin-mediated ERK1/2 intracellular pathway. Integrins are heterodimeric transmembrane receptors, that mediate cell-to-cell and cell-to-extracellular matrix adhesion (ECM) and are considered valuable therapeutic target for the development of new drugs. Particularly, drugs targeting leukocyte integrins, mainly α_4_β_1_ and α_4_β_7_, have proven to be effective for the treatment of inflammatory conditions, including ulcerative colitis, Crohn’s disease, multiple sclerosis (Bamias et al., 2013; Lobatõn et al., 2014; Baiula et al., 2019). Regarding ocular diseases involving inflammation, lifitegrast, an α_L_β_2_integrin antagonist, has been recently approved and is a safe and effective treatment for dry eye disease (DED) (Haber et al., 2019). Lifitegrast binds competitively to the α_L_ integrin subunit preventing the adhesion, migration and proliferation of lymphocytes and the subsequently cytokine production, thereby reducing the symptoms of DED (Kelly et al., 2007; Guimaraes De Souza et al., 2018). Moreover, GW559090, a potent α_4_ integrin antagonist, has been proposed for the treatment of dry eye disease (Krauss et al., 2015) and for Sjögren’s syndrome associated dry eye (Contreras-Ruiz et al., 2016). In addition, the importance of integrins in the pathogenesis of ocular diseases is demonstrated by the possibility to target RGD integrins, such as α_v_β_3_, α_v_β_5_ and α_5_β_1_, expressed on endothelial cells and on RPE. RGD integrins mediate mainly interactions with ECM, and RGD integrin antagonists were shown to prevent the migration of endothelial cells and thereby neovascularization (Hu et al., 2019) and to counteract choroidal angiogenesis and vascular leakage (Yasukawa et al., 2004). Therefore, antagonists targeting RGD integrins may be valuable in ocular diseases involving neovascularization, as in wet AMD.

In a previous study we have reported the pharmacological characterization of DS-70, a potent and selective α_4_β_1_ integrin antagonist, *in vitro* and *in vivo* in a guinea pig model of allergic conjunctivitis (Dattoli et al., 2018). As a selective antagonist of α_4_β_1_, DS-70 prevented integrin-mediated cell adhesion, ERK1/2 phosphorylation and mast cells and eosinophils degranulation *in vitro*. Moreover, DS-70 significantly reduced clinical symptoms of ocular inflammation, α_4_β_1_ expressing cell recruitment to the conjunctiva and pro-inflammatory cytokine and chemokine levels in a guinea pig model of allergic conjunctivitis. Within this frame, in the present study we investigated the effects elicited by DS-70 and other leukocyte integrin antagonists (namely MN27 and SR714) on RPE-lymphocyte crosstalk. MN27 and SR714 are β-lactam derivatives that behave as α_L_β_2_ and α_4_β_1_ antagonists, respectively (Baiula et al., 2016). All three integrin antagonists employed in this study interfere with ARPE-19-Jurkat cells interaction by blocking α_L_β_2_ or α_4_β_1_, they significantly reduce cell adhesion, activation of integrin-mediated intracellular signaling and expression of pro-inflammatory cytokine IL-1β. Moreover, DS-70, MN27 and SR714 significantly prevent apoptosis and necrosis induced in ARPE-19 cells by interaction with Jurkat cells. The disruption of RPE-lymphocyte crosstalk caused by integrin antagonists could be important for the treatment of dry AMD as it reduces RPE cell death and reduced inflammasome activation. These preliminary results provide further support to the hypothesis that α_L_β_2_ or α_4_β_1_ integrin can be considered as valuable drug targets in retinal diseases and that the integrin antagonists employed in the present study could be useful to develop new therapeutic agents useful to fight dry AMD.

The *in vitro* model employed in the current study rely on ARPE-19 cells, a spontaneously immortalized RPE cell line widely used to evaluate RPE function. ARPE-19 cell line represents a simplified cell culture model to study RPE cell functions *in vitro* and displays some differences respect to primary RPE, such as little or no pigmentation, variable morphology and weaker tight junctions; due to these properties ARPE-19 cells rather resemble aged eye or pathological conditions-affected RPE cells (Ablonczy et al., 2011). The current investigation is a preliminary research aimed at studying the involvement of integrins in RPE-immune cells interaction in AMD and the potential use of integrin antagonists within this frame. To confirm data obtained in the current study other *in vitro* cell culture models, including polarized human RPE cells (Khristov et al., 2018) and 3D cultured models (Forest et al., 2015; Shokoohmand et al., 2017; Schnichels et al., 2020) should be further employed. Animal models recapitulating several aspects of AMD are needed to further validate results obtained *in vitro* models. Nonetheless, our findings demonstrate for the first time the involvement of α_4_β_1_ and α_L_β_2_ integrins in the pathophysiology of AMD; thus, pointing at integrin antagonists as promising drug candidates to be further characterized.

In conclusion, integrin antagonists could protect RPE cells from death induced by leukocytes recruited to the retina as they downregulate proinflammatory cytokine expression, reduce ERK1/2 signaling and thereby resulting in the inhibition of the inflammation process. Nevertheless, further studies are needed to better characterize the effects of integrin antagonists in more complex *in vitro* models and in animal models of AMD.

## Data Availability Statement

The datasets generated for this study are available on request to the corresponding author.

## Author Contributions

MB conceived the study, designed the experiments, carried out most of the work, analyzed the data and contributed to write the manuscript. AC and AB contributed to design and perform the experiments. DG and MC designed, synthesized and analyzed the compounds SR714 and MN17; FS, JZ and LG designed, synthesized, and analyzed the compound DS-70. SS designed the study and contributed to write the manuscript. All the authors revised and approved the final version of the manuscript.

## Funding

This work was supported by grants from the University of Bologna RFO 2018, RFO 2019, RFO 2020 to MB and SS; and from a research grant from “Fondazione Cassa di Risparmio in Bologna” (2018/0347).

## Conflict of Interest

The authors declare that the research was conducted in the absence of any commercial or financial relationships that could be construed as a potential conflict of interest.
