# Supplementary material for: Leukocyte Integrin Antagonists as a Novel Option to Treat Dry Age-Related Macular Degeneration
Source: Front Pharmacol. 2021 Jan 29;11:617836. doi: 10.3389/fphar.2020.617836 (PMC7878375; doi:10.3389/fphar.2020.617836)
Supplement: Supplementary file 1 [file datasheet1.docx]

Supplementary Material

# Supplementary Figures

**Supplementary Figure 1.** ARPE-19-Jurkat cells co-culture resulted in increased apoptosis and necrosis. Apoptosis and necrosis were measured by flow cytometry and the results are presented as the percentage of early apoptotic (lower right quadrant) and late apoptotic/necrotic cells (upper right quadrant); see also figure 1 in the manuscript. Representative cytograms are shown.

**Supplementary Figure 2.** Evaluation of ICAM-1 and VCAM-1 expression levels on ARPE-19 and Jurkat cells co-cultured for different time points (0 – 48 h). Representative histograms, obtained by flow cytometry, are shown.

**Supplementary Figure 3.** Analysis of integrin and adhesion molecule expression on ARPE-19 cells evaluated by flow cytometry. Representative histograms are shown.

**Supplementary Figure 4.** Integrin- and adhesion molecule-dependent intracellular signaling was not activated in ARPE-19 cells by mAbs employed in ARPE-19-Jurkat cells co-culture. (A) A representative western blot image is shown and the semiquantitative densitometric analysis of the bands (B) is represented in the graph (mean ± SD of three independent experiments); the amount of pERK1/2 is normalized to that of totERK1/2.

**Supplementary Figure 5.** Integrin antagonists MN27, SR714 and DS-70 (1 - 100 nM) significantly prevented apoptosis and necrosis in ARPE-19 cells induced by 24h of co-culture with Jurkat cells. Cells not co-cultured and treated with the vehicle (DMSO) used to dissolve integrin antagonists were considered as reference (shown as ARPE-19+vehicle in the figure). Apoptosis and necrosis were measured by flow cytometry and the results are presented as the percentage of early apoptotic (lower right quadrant) and late apoptotic/necrotic cells (upper right quadrant); see also figure 6 in the manuscript. Representative cytograms are shown.

**Supplementary Figure 6.** Vehicle employed to dissolve integrin antagonists did not induce apoptosis (A) and late apoptosis/necrosis (B) in ARPE-19 cells, did not alter Jurkat cell adhesion to ARPE-19 cells (C) and did not activate ERK1/2 intracellular signaling in ARPE-19 cells (D-E). ARPE-19 cells were exposed to the corresponding volume of vehicle (DMSO) employed as for 100 nM concentration of integrin antagonists for 24 h. (A-B) Apoptosis and necrosis were measured by flow cytometry and the results are presented as the percentage of early apoptotic cells and late apoptotic/necrotic cells. Values are mean ± SD from four experiments conducted in triplicate using different cell cultures. (C) Jurkat cell adhesion to ARPE-19 cells was measured as described in the methods section. (D) A representative western blot image is shown and the semiquantitative densitometric analysis of the bands (E) is represented in the graph (mean ± SD of three independent experiments); the amount of pERK1/2 is normalized to that of totERK1/2. ***p < 0.001 vs ARPE-19 control cells (Newman-Keuls test after ANOVA).
