# Supplementary material for: Leukocyte Integrin Antagonists as a Novel Option to Treat Dry Age-Related Macular Degeneration
Source: Front Pharmacol. 2021 Jan 29;11:617836. doi: 10.3389/fphar.2020.617836 (PMC7878375; doi:10.3389/fphar.2020.617836)
Supplement: Supplementary file 2 [file datasheet2.docx]

Raw images of each representative western blot; images are displayed in the same order as in the main article.




















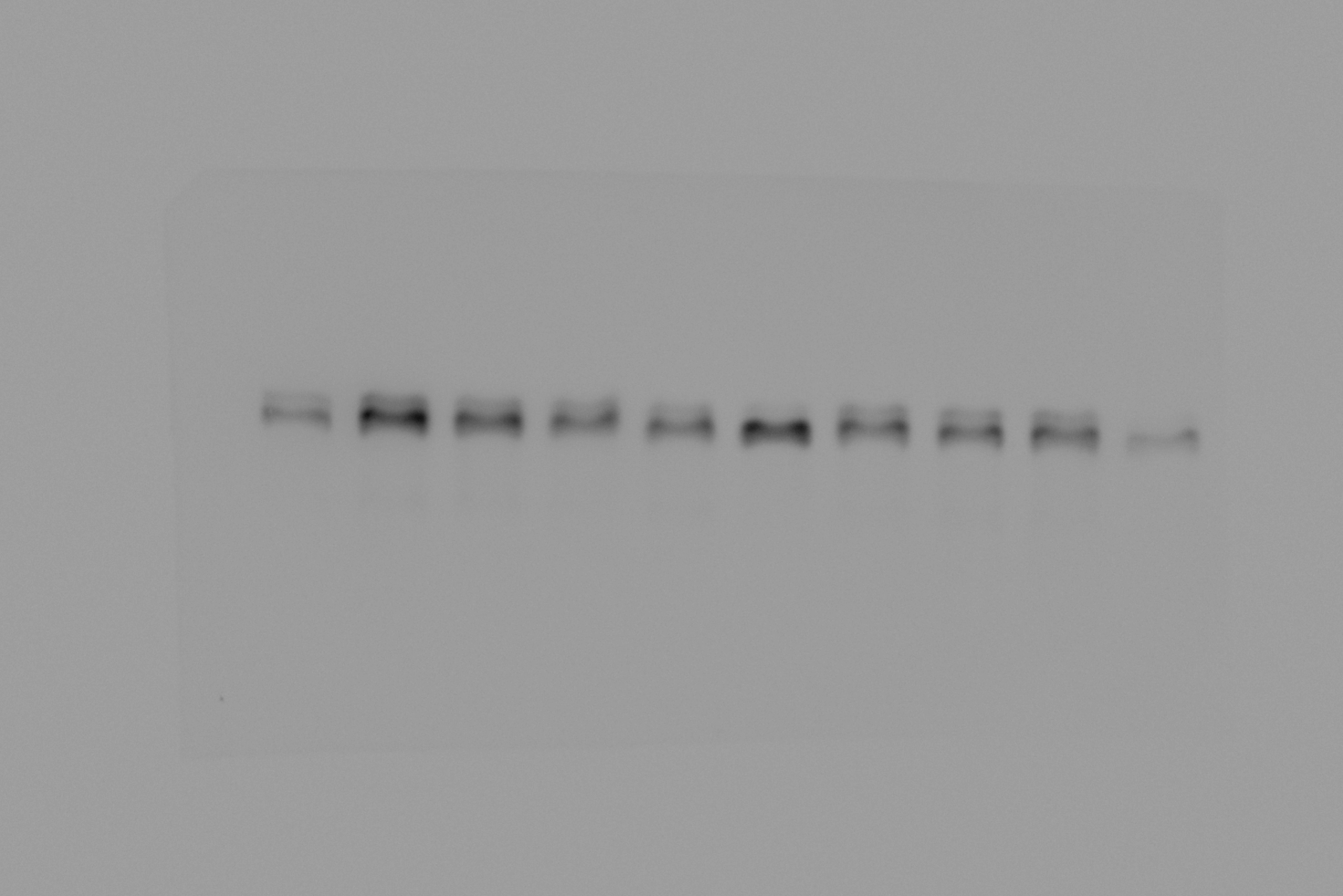


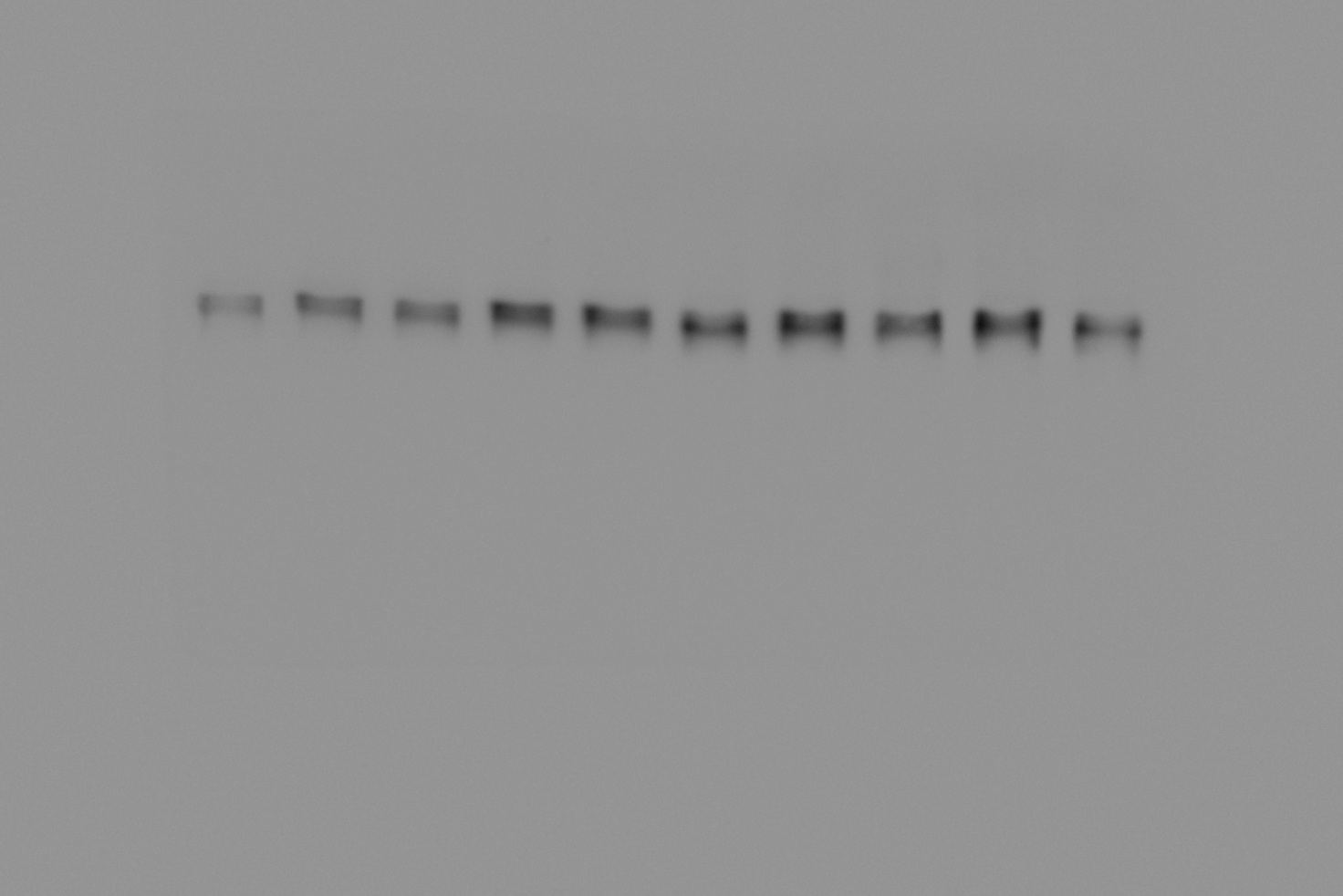








Supplementary Figures
